# Sustained Hippocampal Theta Oscillations Reflect Experience-Dependent Learning in Backward Temporal Order Memory Retrieval

**DOI:** 10.1523/JNEUROSCI.1223-23.2025

**Published:** 2025-05-30

**Authors:** Hongjie Jiang, Jing Cai, Diogo Santos-Pata, Xuanlong Zhu, Zhiyong Deng, Chenyang Li, Ruoxi Luo, Lei Shi, Yudian Cai, Rui Wang, Jiaona Tong, Jia Yin, Shaomin Zhang, Sze Chai Kwok

**Affiliations:** ^1^Department of Neurosurgery, School of Medicine, The Second Affiliated Hospital of Zhejiang University, Hangzhou 310009, China; ^2^Clinical Research Center for Neurological Diseases of Zhejiang Province, Hangzhou 310009, China; ^3^Phylo-Cognition Laboratory, Division of Natural and Applied Sciences, Digital Innovation Research Center, Duke Kunshan University, Duke Institute for Brain Sciences, Kunshan 215316, China; ^4^Shanghai Key Laboratory of Brain Functional Genomics, Key Laboratory of Brain Functional Genomics (Ministry of Education), School of Psychology and Cognitive Science, East China Normal University, Shanghai 200062, China; ^5^ Duke Kunshan University - The First People’s Hospital of Kunshan Joint Brain Sciences Laboratory, Kunshan 215399, China; ^6^The BioBank, Affiliated Kunshan Hospital of Jiangsu University, Kunshan 215399, China; ^7^Department of Pathology, Affiliated Kunshan Hospital of Jiangsu University, Kunshan 215399, China; ^8^Key Laboratory of Biomedical Engineering of Ministry of Education, Qiushi Academy for Advanced Studies, Zhejiang University, Hangzhou 310058, China; ^9^Department of Neurosurgery, China Medical University, The First People's Hospital of Kunshan, Kunshan 215399, China; ^10^Psychological Counseling Center, East China University of Science and Technology, Shanghai 200237, China; ^11^Department of Neurosurgery, Shanghai Tenth People’s Hospital, Tongji University, Shanghai 200072, China

**Keywords:** episodic memory, hippocampus, iEEG, learning, temporal order judgment, theta oscillation

## Abstract

Navigating within our neighborhood or learning a set of concepts requires remembering the relationship between individual items that are presented sequentially. Theta activity in the mammalian hippocampus is related to the encoding and recall of relational structures. However, how theta oscillations are involved in retrieving temporal order information in opposing directionality (forward vs backward) has not been characterized. Here, using intracranial recordings from 10 human epileptic patients of both genders with hippocampal electrodes, we tested the patients with a temporal order memory task in which they learned the spatial relationship among individual items arranged along a circular track and were tested on both forward-cued and backward-cued retrieval conditions. We found that sustained high-power oscillatory events in the hippocampal theta (2–8 Hz) band, as quantified by *P*_episode_ rate, were higher for the backward conditions during the later stage but not in the earlier stage. The theta *P*_episode_ rate results are consistent with the behavioral memory performance and the theta phase to gamma power cross-frequency coupling. Control analyses on change in theta or gamma power and their peak frequencies, aperiodic activity, hemispheric differences, and *P*_episode_ duration confirm that elevated theta rhythmic activity carry specific physiological information with respect to experience-dependent (episodic) learning. In contrast, we observed a stronger effect of forward than backward retrieval for the low gamma (30–70 Hz) *P*_episode_ rate irrespective of stages. Our results revealed how theta oscillations are specifically implicated in the learning process for efficient retrieval of temporal order memories under opposing directionality.

## Significance Statement

While the hippocampus is critical to link events into unitary episodes, the effect of repeated experiences, or experiential learning, on these processes is not entirely clear. We discovered that hippocampal theta oscillation in humans is modulated by repeated experiences, which in turn increases the efficacy of backward-cued memory retrieval of temporal order. This study revealed an important physiological signature characterizing the role of experiences and learning in bidirectional temporal memory retrieval.

## Introduction

The hippocampus is involved in binding items with their spatiotemporal contexts and encoding sequential order information (Squire, 1992; Eichenbaum et al., 1999). Following the discovery of place cells, Mehta et al. (1997) found that the receptive field of a place cell acquires a negatively skewed shape through experience, such that the firing rate is initially low as the animal enters the field but will increase as it exits the arena. Such effects are consistent with place field changes observed during the phase precession effect, where the animal traverses a cell's place field the phase position in which the place cell fires within a theta cycle advances (O’Keefe and Recce, 1993; Skaggs et al., 1996; Tsodyks et al., 1996). Mehta et al. (2000) later proposed that the skewness acquired through experience reflects the directional synaptic strengthening caused by time lag between the pre- and postsynaptic spikes from CA3 to CA1.

In temporal order memory tasks, the encoding of a stimulus could be linked with its “temporal context” in a directional manner, such that this stimulus is associated with those stimuli immediately preceding it more strongly than those following it. By the temporal context model (Howard and Kahana, 2002), retrieved context is an inherently asymmetric retrieval cue and would lead to a widespread advantage for forward recalls compared with backward recalls (Kahana and Caplan, 2002; Yang et al., 2013; Michelmann et al., 2019). Ample behavioral evidence confirmed that forward and backward recalls implicate different retrieval processes (Li and Lewandowsky, 1995; Chan et al., 2009; Liu et al., 2014; Liu and Caplan, 2022). However, how retrieval directionality in memory tests might be modulated by learning remains unknown.

In the case of memory retrieval of temporal order, it is possible that this experience-dependent skewness phenomenon applies on a temporal dimension. Through experience, hippocampal oscillations facilitate temporal sequence memories by transforming rate coding to temporal coding (Mehta et al., 2002). In the human literature, theta oscillations in the hippocampal formation are analogously involved in spatial navigation (Ekstrom et al., 2003, 2005; Liu et al., 2023) and memories (Lega et al., 2012; Solomon et al., 2019), akin to the function of rodent place cells. Indeed, human MTL neurons exhibit a similar form of phase precession during visual sequence learning (Reddy et al., 2021a,b) and item-context binding (Zheng et al., 2022). These suggest that the theta oscillations in the human hippocampus provide a proxy for us to examine the effects of experience-dependent property on memory retrieval. It remains to be elucidated how such experience dependence could affect the efficacy of retrieval directionality (forward vs backward) during memory judgments of temporal order.

In the current study, we recorded extracellular electric potential in the hippocampus postoperatively from epileptic patients following stereoelectroencephalography surgery while they performed a temporal order memory test. Participants viewed through a circular track in virtual reality and then made a relative order memory judgment between two items with respect to a sample. We manipulated the extent of how the participants experienced the virtual reality environment by dividing the experiment into three stages and compared cued judgments of temporal order for forward versus backward sequences. Neurally, we used sustained oscillations in the hippocampus—the *P*_episode_ (Caplan et al., 2001, 2003; Whitten et al., 2011)—to verify our hypotheses. A *P*_episode_ refers to a period during which the power of a specific frequency band exceeds a certain threshold for a sustained duration. As implied by experience-dependent, negatively skewed receptive fields (Mehta et al., 1997, 2000), theta oscillations in the hippocampus should disproportionally reflect higher activation for backward-cued retrieval as the experiment progressed. These effects will be accompanied with a change in memory performance that backward-cued retrieval progressively becomes more accurate. By considering the dynamics between theta phase and gamma power and gamma *P*_episode_ alone, we expected to observe significant theta–gamma coupling during the memory task and the gamma band might have a specific involvement during forward memory retrieval.

## Materials and Methods

### Participant details

Ten medically refractory epilepsy patients were recruited while they were hospitalized for presurgical diagnosis at the Department of Neurosurgery, the Second Affiliated Hospital of Zhejiang University. The patients had been surgically implanted with depth electrodes for diagnostic screening, preceding the surgical treatment (Extended Data Table 1). All patients underwent neuropsychological evaluation and had a normal or corrected-to-normal vision. They were informed about the procedure and provided written consent before participating in the experiment. The study was approved by the Human Research Ethics Committee of the Second Affiliated Hospital of Zhejiang University School of Medicine (IR202001335).

### Experimental design and statistical analyses

#### Experimental paradigm

**Figure 1.**
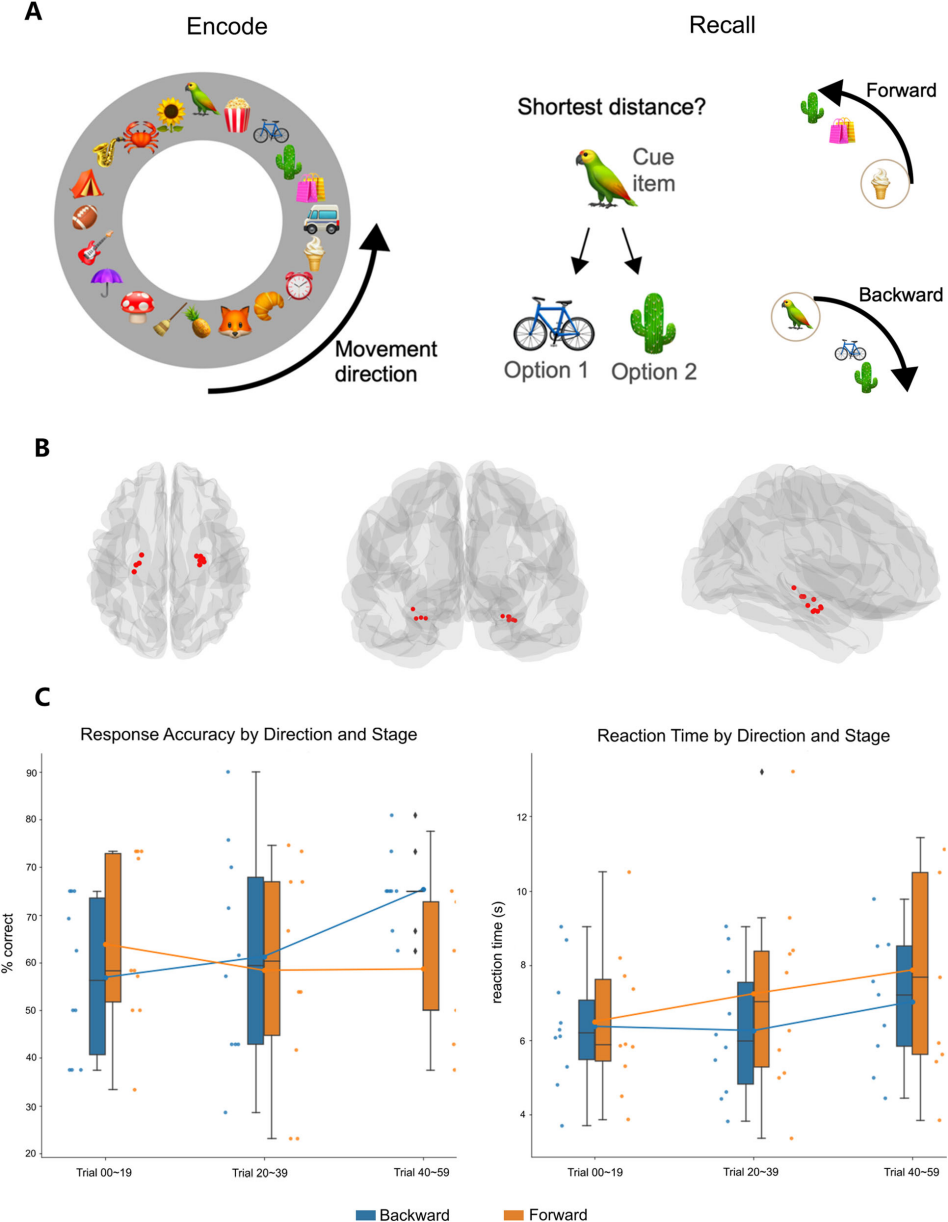
***A***, Illustration of the virtual navigation task paradigm. Left, Birdview layout of the circular navigation path (not shown to participants throughout encoding). Right, Example of a cued-distance judgment task during recall. Experimental condition: recall directionality (forward/backward), depending on the order in which items were encountered during passive navigation. ***B***, Electrode locations in the hippocampus from individual patients (*n* = 10) in MNI space coordinates by top, back and right views. ***C***, Left, Memory task accuracy; Right, memory task reaction time. Each dot represents a single participant's performance at early, middle, or late stage of the task, under forward or backward condition. Boxes denote the 25th to 75th percentile and the median line. Whiskers extend 1.5 times the interquartile range from the edges of the box. The lines on the boxplots indicate the means for each of the conditions.

Participants performed a passive navigation task in a virtual reality (VR) environment within a circular track at a constant speed. Patients performed the task on a laptop while sitting in a comfortable position in their hospital bed at a distance of ∼50 cm from the screen. The VR application was created using the Unity3D game engine (Unity Technologies). Four 3D models of buildings placed on the outer part of the track served as global environmental landmarks during navigation. Within the track, a set of local cues composed of everyday-life objects, obtained from the “texture city prefabs” library for Unity3D (https://unity.com/), were randomly selected and placed at uniform distances along the navigational path (see Fig. 1*A* for a birdview of the virtual environment). The participants viewed the track from a first-person perspective. The starting point position in each trial was randomized but the order of items stayed the same and participants always faced the same direction. Sixty trials in total were completed by each patient, lasting ∼40 min.

#### Navigation and memory task

Each trial started with a self-paced blank screen with a black fixation cross in the center alerting the subject of the beginning of a new trial. At each trial, the number of local landmarks placed in the surroundings of the circular track and the locomotion speed of the virtual avatar varied accordingly with the experimental condition. The number of visual cues placed on the track was set to either 50, 100, or 150 landmarks (determining the diameter of the circular track), and the speed of locomotion was set as 19.9, 26.7, or 32.7 deg/s, for the low, medium, and high information density conditions, respectively. Each navigational lap contains 20 items and the mean and standard deviation of the number of laps per trial are 1.43 ± 0.34 (Movie 1). Details for the encoding stage and part of the related data were reported previously (Santos-Pata et al., 2022).

At the end of the navigation, a blank screen appeared for 4 s, participants were then probed with a cue item and asked to indicate which of the two items was closer to the cue during navigation. For the memory judgment of relative temporal order, we operationally defined two levels of this condition by the order in which the cue and choices were encountered: forward or backward search (Fig. 1*A*). Note that these two option items were always in the same direction to the cue, within one quadrant of the circular track (i.e., <5 items apart). The trial presentation order was randomized across participants. Our analysis focused on the memory recall stage.

#### Electrophysiological recordings and electrodes localization

The location of the electrodes was established only for clinical reasons using a stereoelectroencephalography (sEEG) approach. Targeted regions varied across patients depending on their clinical assessment, but in all participants recruited for this study, they included the hippocampus in the left (*n* = 4) or the right hemisphere (*n* = 6). After coregistering pre- and postelectrode placement using MR scans and CT whole-brain volumes, we confirmed 10 pairs of contacts located in the hippocampus (Fig. 1*B*). Locations of the electrodes in native space were finally converted to the standard Montreal Neurological Institute (MNI) space using 3D Slicer (https://www.slicer.org) and BrainX3. Recordings were made using a standard clinical EEG system (EEG-1200C, Nihon Kohden) at a sampling rate of 2,000 Hz. A unilateral implantation was performed in all patients, with each of them having 8–11 intracranial electrodes (Sinovation; diameter, 0.8 mm; 8–16 contacts; 2 mm length; 1.5 mm spacing between contact centers) stereotactically inserted with robotic guidance (Sinovation, Medical Technology).

#### Event-related field potential and preprocessing

When more than one contact was available, the electrode with higher delta-theta power ratio was selected. By selecting electrodes with the strongest delta-theta power ratio, we prioritized our selection on signals with high-power oscillatory events in the hippocampal theta band. In the rodent, some previous studies have also adopted this delta/theta ratio approach (Santos-Pata et al., 2023). In this context, we aimed to target electrodes associated with relevant memory-related processes [Pacheco Estefan et al. (2019) identifying coupling of hippocampal delta and theta oscillations]. A bipolar montage was used offline to mitigate any effect of volume conduction or any confounds from the common reference signal. Bipolar signals were derived by differentiating electrodes pairs, the contact of interest, and one contact from adjacent white matter identified anatomically, of recorded and not rejected channels within the same electrode array. The continuous iEEG signals at the selected electrodes were first bandpass filtered between 1 and 200 Hz using a two-way, zero phase-lag, finite impulse response filter to prevent phase distortion (*mne.filter.filter_data* function). To remove power line contamination, we applied a notch filter at 50 Hz and harmonics with a 2 Hz bandwidth (*mne.filter.notch_filter* function). Stimulus-triggered TTL pulses were also recorded with the iEEG data for synchronization with task events. For each patient, the hippocampal event-related field potentials were computed by averaging the preprocessed signals from the selected contact points locked to the memory retrieval stage onset.

#### Power spectrum fit and peak frequency analysis

The power spectrum density was computed using the Welch method (using the *scipy.signals.welch* function) and fitted to the 1/*f* background spectrum in a log-log space to show frequency dependence of bands during recall task where oscillatory power exceeded 1 standard deviation over the 1/*f* background. We also calculated the number of oscillatory activities whose estimated peak frequency falls in 1–15 Hz and 25–75 Hz respectively using the FOOOF library (http://www.fooof-tools.github.io) and detected dominant frequencies during memory retrieval for each subject where counts of peak frequencies exceeded 2 standard deviations of the mean numbers of peak frequencies detected within the frequency range of interests. We also applied the FOOOF method to characterize the aperiodic components and model fit. We computed the aperiodic fit for each PSD split by frequency band and obtained the slope and *y*-intercept (aperiodic components exponent and offset) for each PSD computed per subject. We performed analyses to check whether there were any effects of the experimental procedure on the level of aperiodic activity (exponent and offset).

#### *P*_episode_ rate and *P*_episode_ duration analysis

We utilized the oscillatory episode detection algorithm to identify high-power rhythmic activity. We used the measure *P*_episode_, which is defined as the proportion of time during which oscillations at a given frequency were present (Caplan et al., 2001, 2003; Caplan and Glaholt, 2007; van Vugt et al., 2007; Whitten et al., 2011). We operationally defined an oscillatory episode at a frequency of interest, f*, as a duration that exceeded a time threshold, DT, during which power at frequency f* was greater than a power threshold, PT. We selected the values of PT and DT as follows. We applied a Morlet wavelet transform to the raw data traces to move to the frequency domain. We used 28 frequency steps logarithmically sampled in the range of 1–76 Hz to preserve the relative bandwidth. The wavelet transform provides us with wavelet power as a function of time at each frequency of interest. To determine the value of PT at each recording site for each frequency, we assumed that the background spectrum was “colored noise,” with the form of Af-α. We fitted the theoretical Power (*f*) = Af-α function to the actual power spectrum over the 1–76 Hz range at each electrode. We then took the fit value at the frequency of interest, f*, as the mean of the *χ*^2^(2) distribution of wavelet power at that frequency and set PT at that frequency to the 95th percentile of the cumulative distribution of this fit *χ*^2^(2) function to exclude 95% of the background signal. The duration threshold, DT, at frequency f* was set to three cycles [i.e., 3(1/f*)] to eliminate artifacts and physiological signatures that were nonrhythmic. *P*_episode_(*f*) were originally defined as the total time during which episodes occurred at frequency *f* divided by the total trial time. We adapted the measurement of *P*_episode_(*f*) by calculating the number of oscillatory events per second across the frequency bands of interest (2–8 Hz for theta and 30–70 Hz for gamma), namely, the rate of *P*_episode_.

We also examined the corresponding effects by using the proportion of time (*P*_episode_ duration) for the *P*_episode_ data. We note however that using the proportion of time for the *P*_episode_ might not be the same as the *P*_episode_ rate. *P*_episode_ are intrinsically dependent on a temporal metric (frequency). One of the criteria for a *P*_episode_ is that it must sustain power above *X* std, for *Y* number of cycles, of a given frequency (usually *Y* is set at 3 cycles). For instance, when we consider theta in the 2–10 Hz range. One *P*_episode_ in 2 Hz freq requires 1.5 s to be considered as an episode, whereas a *P*_episode_ in the 10 Hz freq requires 0.3 s to be considered as an episode. Because the trial duration is fixed, irrespective of the *P*_episode_ frequency, the variability in theta (lower or higher) will introduce biases. In contrast, by *P*_episode_ rate, events become discrete and their overall duration might induce a smaller bias for the interpretation. We should also be cautious that *P*_episode_ are not bound to one unique frequency as they often appear to have a blob-like shape—they can occupy a range of subfrequencies (e.g., 3.5–5 Hz). To deal with their characterization, one option would be to consider *P*_episode_ on a frequency-by-frequency basis (1, 2, 3 Hz…). In that case, some of the episodes would overlap with each other and episode duration would be counted multiple times. *P*_episode_ rate, on the other hand, characterizes time points where multiple events are detected across frequencies of interest as one event. In other words, *P*_episode_ rate can turn events to be discrete, thereby reducing bias due to varied trial duration and/or multiple counting of the episodes.

#### Generalized linear mixed models

We built linear mixed models (LMMs) with package lme4 (Bates et al., 2015) in R (R Core Team, 2012) analyzing the effect of directionality condition and stage of the task on rates of *P*_episode_ and *P*_episode_ duration for theta and gamma oscillations, respectively. Behaviorally, we ran two separate generalized linear mixed models with package car (Fox and Weisberg, 2011) to analyze the effect of directionality condition and stage of the task on response accuracy (binomial family, logit link function) and reaction time (inverse Gaussian family, inverse link function). For all models, we adopted a 2 × 3 design where two levels of recall directionality (forward vs backward) and three levels of learning phase (early vs middle vs late stage of the task) were entered as fixed effects, with intercept for subjects entered as random effects to account for individual differences in overall task performance and missing data points. Type 3 likelihood ratio tests comparing the full models against corresponding null models were used to obtain *p* values.

#### Phase–amplitude coupling analysis

Hippocampal phase–amplitude coupling (PAC) was computed for signals recorded during the memory recall periods. We applied a Fourier Transformation to the hippocampal local field potentials (LFPs), which were sampled at a rate of 2,000 Hz. This transformation decomposes the LFP data into their constituent frequency components, providing us with the basis for subsequent PAC analysis. Specifically, we focused on extracting the theta oscillations (2–8 Hz) to capture the phase and gamma oscillations (30–70 Hz) for the amplitude component (Tort et al., 2010; Lisman and Jensen, 2013). The computation of PAC was performed through the Kullback–Leibler divergence method (Pérez-Cruz, 2008). This method involves the calculation of the modulation index (MI), quantifying the statistical dependency between phase and amplitude. Mathematically, MI is expressed as follows:

MI=ΣiΣjP(θi,Aij)*log2[P(θi|Aij)/P(θi)],

where *θi* represents the phase angle at time point *i*, Aij denotes the amplitude at time point *j*, and *P*(*θi*, Aij), *P*(*θi* | Aij), and *P*(*θi*) are the joint, conditional, and marginal probability distributions, respectively. MI measures the difference between the joint distribution of phase and amplitude and the product of their marginal distributions, indicating the extent of coupling. To establish statistical significance, we conducted hypothesis testing, employing the Bootstrap-Coupled Estimation (BCE) method to assess statistical significance in our data analysis (Ho et al., 2019).

## Results

### Memory performance

In terms of response accuracy, a significant interaction between the two fixed effects was found [*F* (df = 2) = 6.26, *p* = 0.044], highlighting a diverging pattern of change in performance across stages of the task by recall directionality condition. Estimated marginal means (EM) with 95% confidence interval further suggested that at early stage of the task, response accuracy on forward trials (EM = 0.644, 95%CI = [0.540, 0.735]) was higher than backward trials (EM = 0.577, 95%CI = [0.459, 0.686]); however at late stage of the task, response accuracy on backward trials (EM = 0.773, 95%CI = [0.671, 0.851]) became higher than forward trials (EM = 0.598, 95%CI = [0.475, 0.709]; Fig. 1*C*).

No significant effect of interaction was found on reaction time [*F* (df = 2) = 1.17, *p* = 0.558]. There was however a significant fixed effect of recall directionality [*F* (df = 1) = 6.49, *p* = 0.011], where backward trials were faster than forward trials, as well as a fixed effect of task stage [*F* (df = 2) = 9.22, *p* = 0.010], where responses were slower toward the end of the experiment (Fig. 1*D*).

### Frequency dependence and peak frequencies during recall

Before looking into the effects of experimental manipulations, we first demonstrated the frequency dependence of the recall task across participants. Focusing on the theta (Extended Data Fig. 1*A*) and low gamma frequencies (Extended Data Fig. 1*B*), we fitted the power spectrum density to the 1/*f* background spectrum in a log-log space and identified the frequency bands where oscillatory power exceeded 1 standard deviation over the 1/*f* background (Extended Data Fig. 1, left subplots, gray regions). Moreover, we calculated the number of oscillatory activities and detected subject-specific dominant peak frequencies (Extended Data Fig. 1, right subplots, histograms). Both measures consistently revealed prominent oscillations during recall at the theta band and low gamma band, which gave us an understanding of the frequency dependence for the task. After taking into account each subject's variation, this result guided us to choose the frequency band of 2–8 Hz (theta) and 30–70 Hz (gamma) for the *P*_episode_ analysis and power and peak frequency analyses.

### *P*_episode_ rates

**Figure 2.**
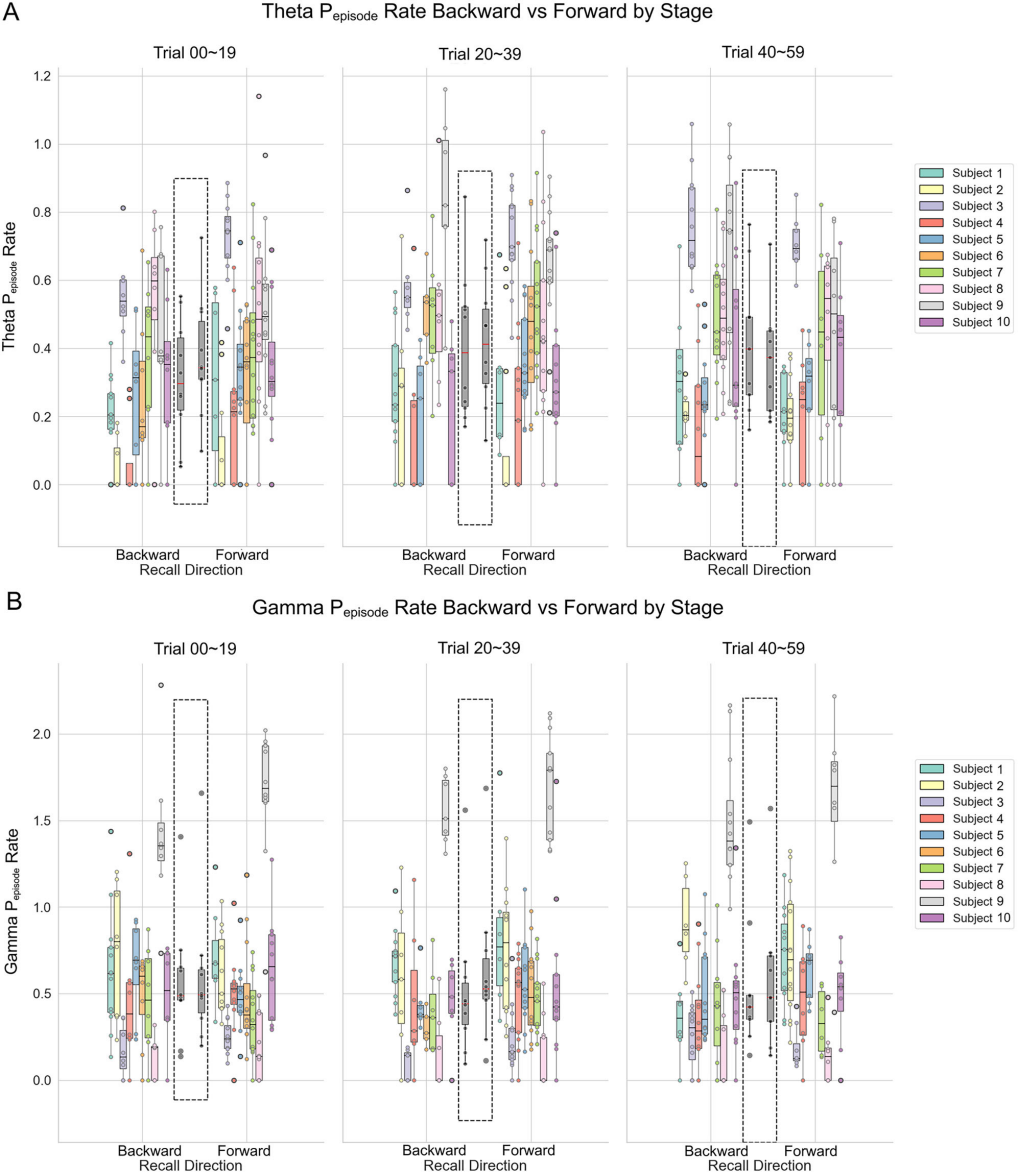
*P*_episode_ rates by conditions for theta and low gamma activity. ***A***, Theta *P*_episode_ rates organized by directionality and stage conditions. There is a directionality by stage interaction. ***B***, Gamma *P*_episode_ rates organized by directionality and stage conditions. There is a main effect of directionality irrespective of stage. Each color denotes an individual patient. Boxes denote the 25th to 75th percentile and the median line. Whiskers extend 1.5 times the interquartile range from the edges of the box. All-subject group averages per condition are displayed in the center of each panel (marked by dashed line rectangles).

Neural oscillations are often analyzed in terms of both their power and phase, and *P*_episode_ focus on periods where there is an elevated power of these oscillations. In line with memory accuracy, a significant interaction effect [*F* (df = 2) = 6.90, *p* = 0.032] was revealed where the rates of theta *P*_episode_ during forward trials (EM = 0.374, 95%CI = [0.266, 0.482]) was higher than those during backward trials (EM = 0.310, 95%CI = [0.200, 0.421]) at the early stage of the task, followed by a reversed pattern at the late stage of the task when the rates of theta *P*_episode_ became higher for backward trials (EM = 0.418, 95%CI = [0.308, 0.527]) than those during forward trials (EM = 0.369, 95%CI = [0.258, 0.480]; Fig. 2*A*). We also found a significant fixed effect on task stages, where theta *P*_episode_ gradually increased along stages of the task [*F* (df = 2) = 11.50, *p* = 0.003]. For comparison, we performed a corresponding set of *P*_episode_ analysis on the low gamma band (30–70 Hz). We found no significant fixed effect of task stage or interaction but only a fixed effect on recall directionality *P*_episode_ [*F* (df = 1) = 5.59, *p* = 0.018], where low gamma *P*_episode_ was higher for forward trials throughout all stages (EM = 0.595, 95%CI = [0.362, 0.828]) than that for backward trials (EM = 0.538, 95%CI = [0.304, 0.771]; Fig. 2*B*).

### *P*_episode_ duration

**Figure 3.**
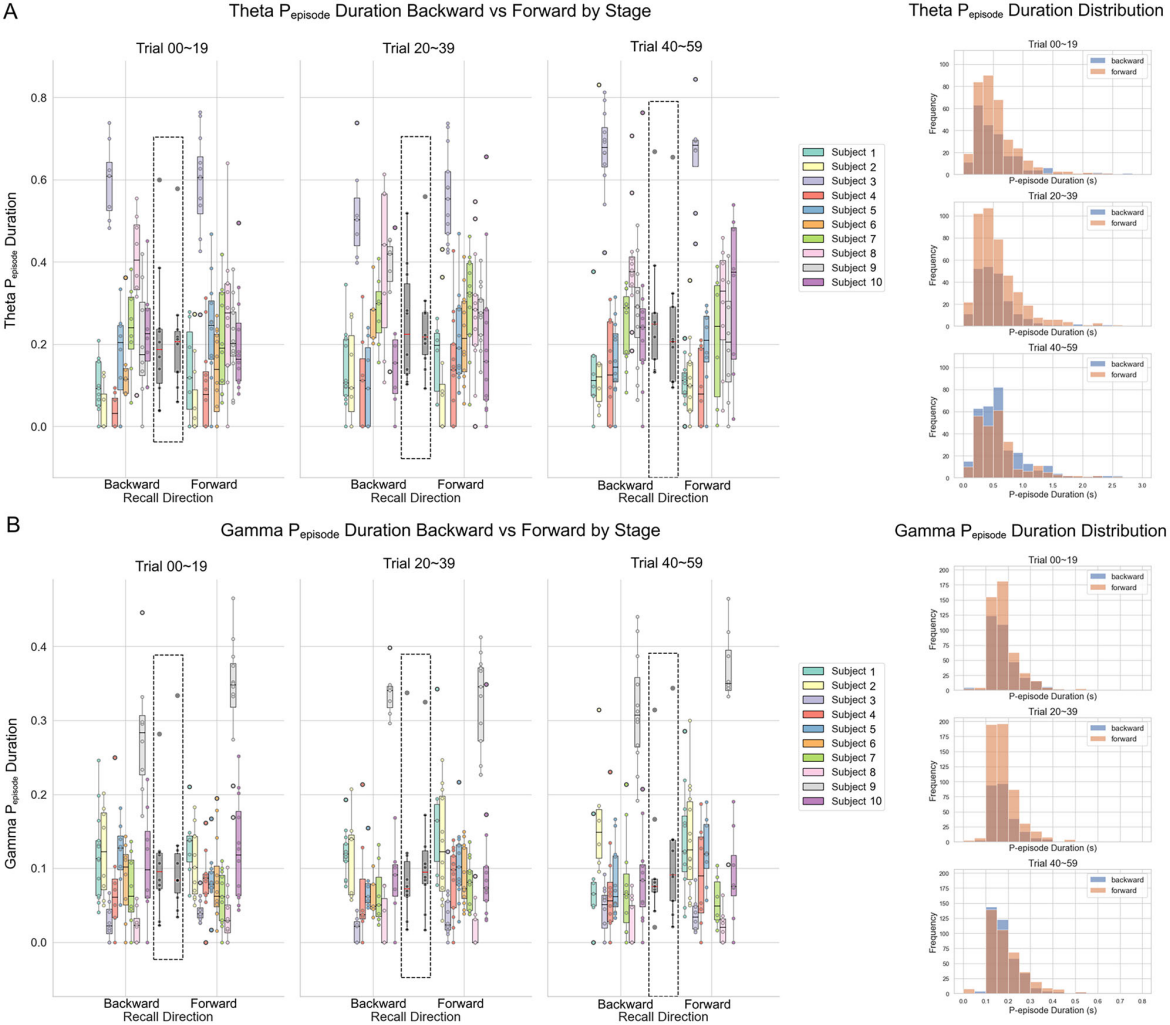
*P*_episode_ duration by conditions and distributions for duration of *P*_episode_ (event length, in s) for theta and low gamma. ***A***, Theta *P*_episode_ duration organized by stage and directionality conditions (left). All-subject group averages per condition are displayed in the center of each panel (marked by dashed line rectangles). Distributions for duration of the *P*_episode_ by stage and directionality conditions (right). ***B***, Gamma *P*_episode_ duration organized by stage and directionality conditions (left). All-subject group averages per condition are displayed in the center of each panel (marked by dashed line rectangles). Distributions for duration of the *P*_episode_ by stage and directionality conditions (right).

Moreover, we examined the corresponding effects by using the proportion of time for the *P*_episode_ data, or the so-called *P*_episode_ duration. We ran an LMM on the duration for the *P*_episode_ data and obtained a significant fixed effect of task stage on theta *P*_episode_ duration [*F* (df = 2) = 10.54, *p* = 0.005], but the interaction effect was not replicated [*F* (df = 2) = 1.28, *p* = 0.528; Fig. 3*A*, left]. For the proportion of *P*_episode_ time for gamma, the results were consistent with *P*_episode_ rate, where we only see a significant fixed effect of directionality on gamma *P*_episode_ duration [*F* (df = 1) = 5.70, *p* = 0.017; Fig. 3*B*, left]. For visualization purposes, we produced the distributions for the duration of the *P*_episode_ separately for theta and gamma bands for each recall directionality and task stage condition (Fig. 3*A*,*B*, right).

### Relationship between theta oscillations parameters and behavioral aspects

**Figure 4.**
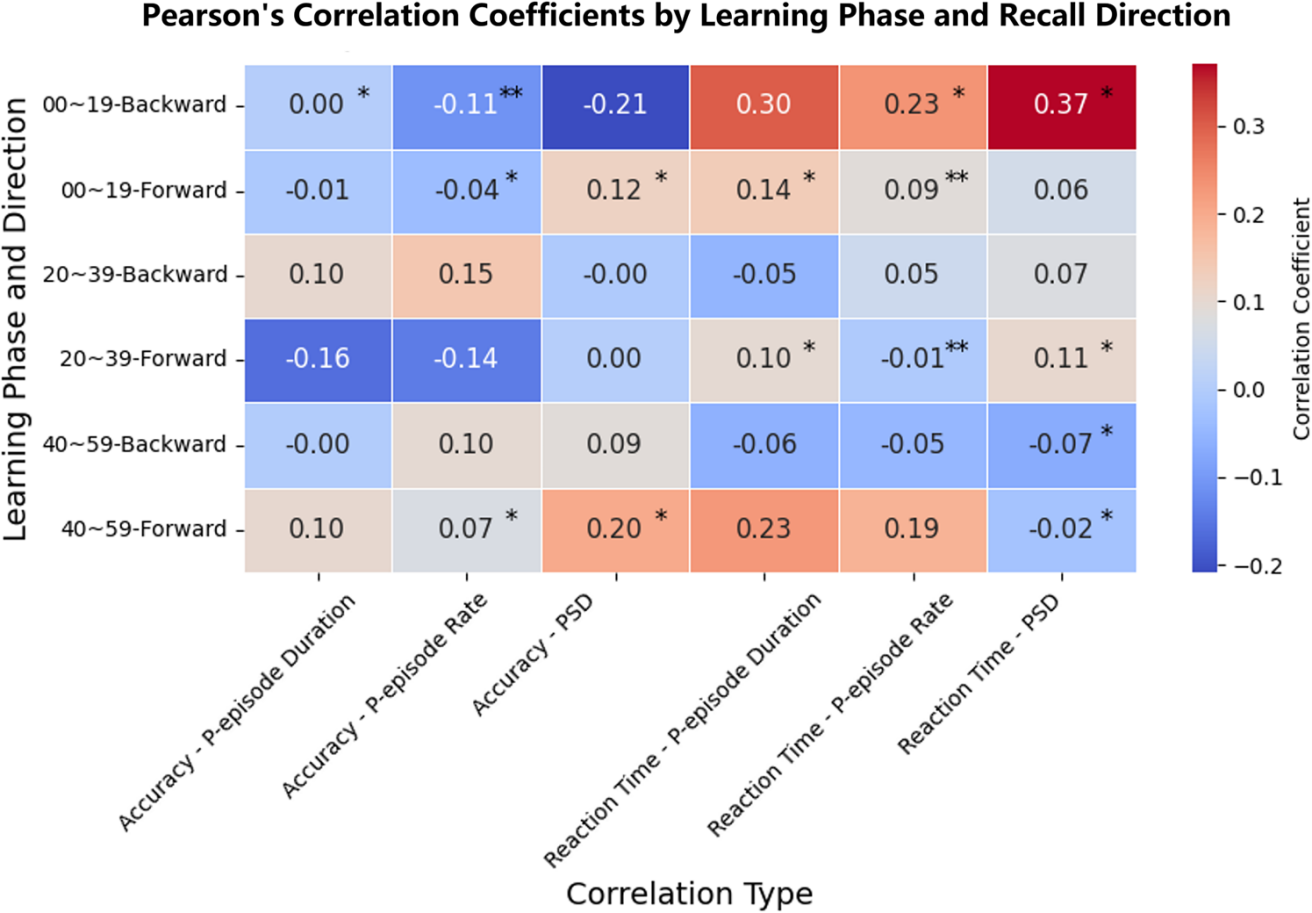
Pearson’s correlation coefficient matrix for subject-wise accuracy or mean reaction time with either their theta *P*_episode_ rate or theta *P*_episode_ duration or theta power per stage and directionality conditions across participants. Significant correlation coefficients are denoted with asterisks (* indicates *p* < 0.05; ** indicates *p* < 0.01). The total number of subjects was 10 per condition; 6 conditions (2 directions × 3 learning phases) included.

By considering the relationship between theta oscillations parameters and functional/behavioral aspects, we found that the three theta parameters are correlated with mean reaction time irrespective of conditions (theta *P*_episode_ rate: *ρ* = 0.27, *p* = 0.040; theta *P*_episode_ duration: *ρ* = 0.34, *p* = 0.009; theta power: *ρ* = 0.457, *p* < 0.001) but not with accuracy (all *p*s > 0.500). On top of that, for completeness, we calculated all the pairwise Pearson’s correlation coefficients between either subject-wise accuracy or mean reaction time with either their theta *P*_episode_ rate or theta *P*_episode_ duration or theta power per stage and directionality conditions across the participants (Fig. 4). Specifically for the relationship between our key parameter (i.e., theta *P*_episode_ rate and theta *P*_episode_ duration) and mean reaction time in the backward condition, there appeared to be a change in correlational direction across learning stages (from positive *ρ* = 0.23, *p* < 0.05 to negative *ρ* = −0.05, *p* < 0.05; theta *P*_episode_ duration: from positive *ρ* = 0.30, *p* < 0.05 to negative *ρ* = −0.06). These results further indicate the relationship between theta oscillations and certain functional aspects in our task.

### Phase–amplitude coupling results

**Figure 5.**
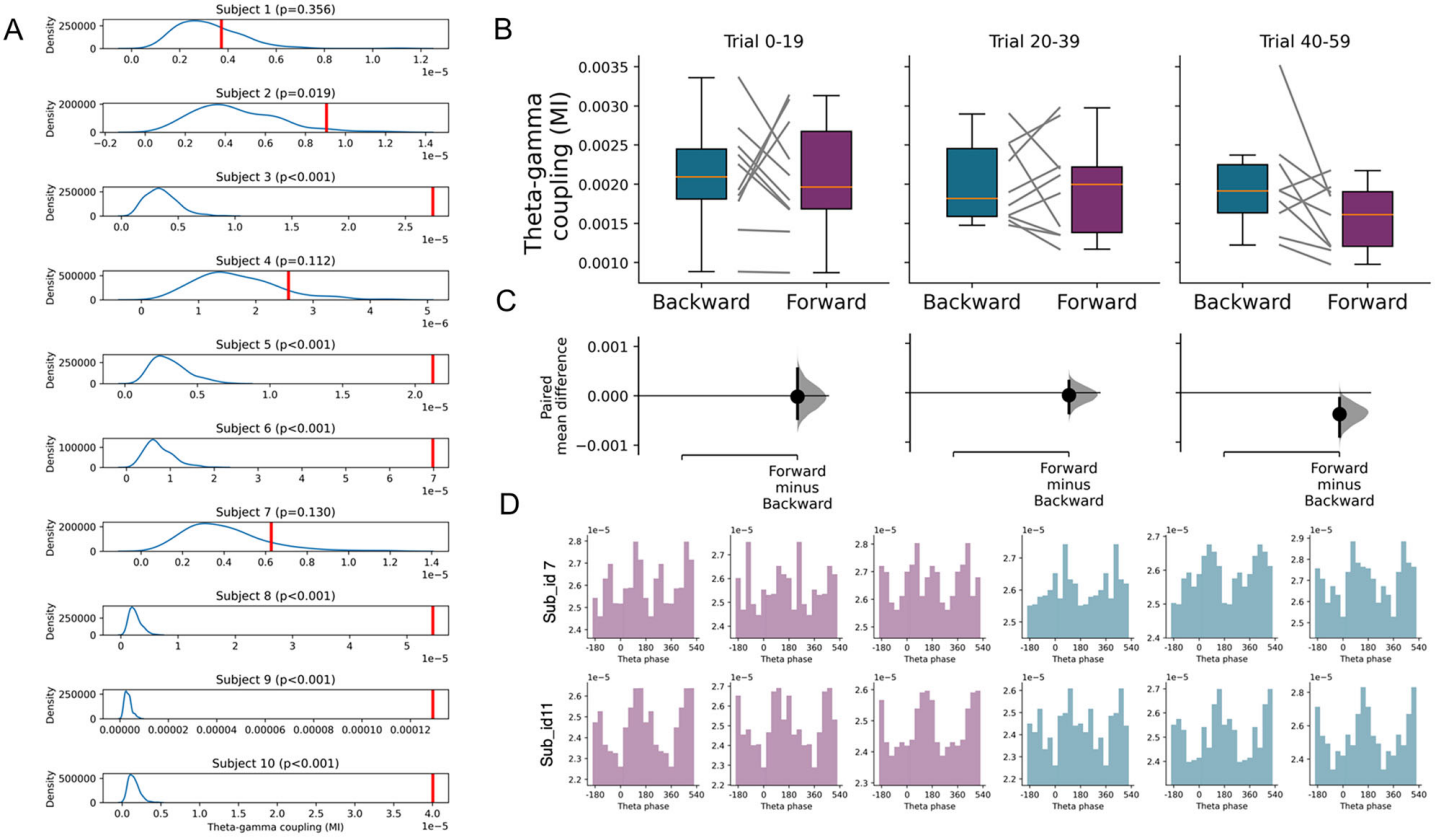
Theta phase to gamma power cross-frequency coupling analysis by experimental conditions. ***A***, Theta–gamma coupling for each subject (red vertical line) and tested against permuted distributions (blue density distribution). *p* values are reported per subject's comparison. ***B***, Comparing theta–gamma coupling scores (modulation index, MI) between directionality conditions for the three stages. ***C***, The mean differences between forward and backward conditions are shown for the early, middle, and late stages. ***D***, Subjects 7 and 11 are shown as two examples. The three subplots (from left to right) refer to early, middle, and late stages.

In the hippocampal area, the interplay of theta and gamma rhythms often plays functional roles. It is important to examine the correlation or theta–gamma coupling. One way to ascertain whether the dynamics of theta and gamma power are correlated in each participant is to run a theta phase to gamma power cross-frequency coupling analysis (PAC analysis). We obtained theta–gamma coupling for each subject and tested against permuted distributions (7/10 participants with *p* < 0.05; Fig. 5*A*). The results indicate that the dynamics of theta phase and gamma power are coupled in each subject during the memory task (Fig. 5*A*,*B*, with two example participants shown in Fig. 5*D*). Furthermore, the mean difference between forward and backward conditions for the three stages are the following: 0.016 × 10^−4^ (early), 0.046 × 10^−4^ (mid), and 0.428 × 10^−4^ (late; Fig. 5*C*). We found a marginal effect of stage (*p* = 0.06) but no main effect of directionality (*p* = 0.50) and no stage × directionality interaction (*p* = 0.44). Despite the lack of an interaction, we tested for statistical significance between backward and forward conditions for each stage and found that only the difference at the late stage shows a trend toward significance (late, *p* = 0.06, with numerically stronger coupling in the backward condition than forward condition), but not with the other two stages (early, *p* = 0.92; mid, *p* = 0.78). This stronger coupling for the backward condition in the late stage is somewhat consistent with the findings with the theta *P*_episode_ rate.

### Change in theta/low gamma power and peak frequencies

**Figure 6.**
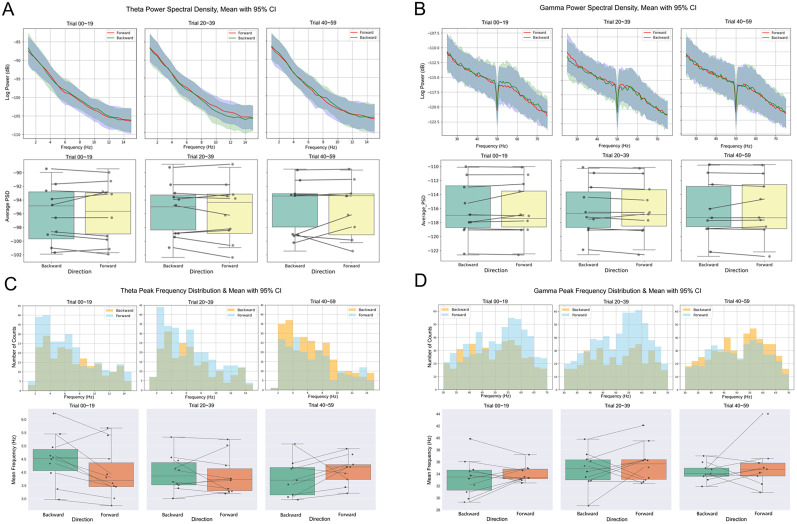
Power spectral densities and distributions of peak frequencies for theta and low gamma activity. ***A***, Theta oscillatory profile in terms of power spectral densities (PSD) for backward and forward recall direction per learning stage (top) and averaged power amplitude comparison by experimental conditions (bottom). ***B***, Low gamma oscillatory (PSD) profile for backward and forward recall direction per learning stage (top) and averaged power amplitude comparison by experimental conditions (bottom). ***C***, Within the theta band, distributions of peak frequencies (top) and mean peak frequencies (bottom) by experimental conditions. ***D***, Within the low gamma band, distributions of peak frequencies (top) and mean peak frequencies (bottom) by experimental conditions.

Oscillations can be characterized not only by their occurrence but also by their power and peak frequencies. For that reason, we computed the power and peak frequencies for each of the conditions and tested for their main effects and interactions, separately for theta and low gamma bands. The power was analyzed by computing the averaged PSD for each condition along learning stage (Fig. 6*A*,*B*, top) and the average theta power was compared for both direction conditions (Fig. 6*A*,*B*, bottom). Two-way ANOVAs were conducted to examine the effects of learning stage and directionality on the power within the theta band (2–8 Hz) and within the low gamma band (30–70 Hz), respectively. There were no significant main effects or interactions (all *p* > 0.05) in either of them. Secondly, we obtained the distribution of peak frequencies within the theta band (2–8 Hz) and within the low gamma band (30–70 Hz), for each of the experimental conditions (Fig. 6*C*,*D*, top), and tested for the factors’ main effects and their interaction between stage and directionality (Fig. 6*C*,*D*, bottom). Two-way ANOVAs were conducted to examine the effects of learning stage and direction on peak frequency within the theta band and within the low gamma band. There were neither significant main effects nor interaction (all *p* > 0.05). Since the interactional terms of both of these ANOVAs were not significant (*p*s > 0.05), we did not further conduct post hoc corrections to determine which specific groups differ from each other.

**Movie 1.** A movie showing the experimental paradigm. [View online]

### Control analysis on aperiodic activity

Aperiodic neural activity is known to link to certain cognitive processes (Zhang et al., 2023; Yan et al., 2024). We thus performed analyses to check whether the level of aperiodic activity (exponent and offset) for each PSD computed might be linked to our experimental procedure. We found that there is no significant difference between the two frequency bands (Exponent: mean difference = 0.171, permutation test *p* =0.219; Offset: mean difference = 0.211, permutation test *p* = 0.265). Moreover, we tested for differences in the aperiodic components (i.e., offset and exponent) for the two experimental conditions of directionality (backwards and forward) with one-way ANOVAs separately for theta and low gamma bands. We found that there are no differences in all four comparisons, all *F*s < 1. These analyses suggest that the effects of the experimental procedures/conditions do not affect the aperiodic activity. In sum, we can see that bands and the directionality factor do not differ significantly in terms of their aperiodic fit exponent or offset, reassuring that specificity of our findings are not likely due to aperiodic activity. These aperiodic results are helpful to ensure that our main results and the PAC outcomes are primarily attributed to periodic oscillatory activity.

### Control analysis on differences between left/right hemispheric signals

Among the 10 recording sites in the hippocampus, we have four and six electrodes in the left and right hemispheres, respectively. We added the electrodes’ hemisphere as an additional factor and re-ran the LMM on the main analyses on the rates of *P*_episode_ for theta band. We found that the hemisphere as a fixed effect factor did not change our main results [*F* (df = 1) = 0.438, *p* = 0.508]. This suggests there were no discernable differences between the left and right hippocampal signals.

## Discussion

We investigated the role of hippocampal theta activity in recall directionality using a relative order judgment task following encoding in a virtual circular track. The sustained high-power oscillatory events in the theta band increased for the backward-cued than forward-cued recall conditions during the later stage, compared with the earlier stage of the task. This suggests that learning results in experience-dependent changes in the neural signal in the hippocampus.

Across a number of control analyses, we made our key finding specific as the learning effect is not reflected with other related parameters including change in theta power, or their peak frequencies, *P*_episode_ duration of the data, or differences in aperiodic activity. These imply that high-power rhythmic activity (i.e., exceeding a power threshold and exceeding a time window threshold, van Vugt et al., 2007; Whitten et al., 2011) in the hippocampus carries important and unique physiological information with respect to experience-dependent learning for backward-cued memory order retrieval. This experience-dependent effect also partially manifested itself via the theta phase to gamma power cross-frequency coupling, implying a role played by an interplay between theta dynamics and gamma power (Lisman and Jensen, 2013). In light of observations that hippocampal place fields are positively skewed in their initial states, before gaining a negatively skewed shape through learning or experiences in both rodents (Mehta et al., 2000) and humans (Rutishauser et al., 2010; Reddy et al., 2021a,b), our finding extended this possibility to memory retrieval.

The hippocampus codes for abstract concepts and memories in some similar way as coding for space (Constantinescu et al., 2016; Bellmund et al., 2018; Solomon et al., 2019). This notion aligns with recent work on hippocampal theta that are indicative of links between navigational space (Chen et al., 2021) and information search through mental spaces, such as temporal and semantic clustering in memory recall (Sakon and Kahana, 2022; Sakon et al., 2024). Many recent studies have shown that the hippocampus is involved in nuanced memory processes such as replay of items (Liu et al., 2019; Nour et al., 2021) or time (Zhu et al., 2025), recall of sequence of naturalistic events (Lehn et al., 2009), and representing multiplexed event and temporal structures (Liu et al., 2022). These processes might be related to other findings on time-encoding cells (Umbach et al., 2020), temporal context cells (Bright et al., 2020; and see also Zuo et al., 2023), and temporally periodic cells (Aghajan et al., 2023 ). In comparison, our present finding on changes in the mammalian hippocampal theta signal over repetitions is important in two aspects. Firstly, the sustained oscillations suggest that differential contextual reinstatement during memory retrieval is dependent on the cued directionality (cf. Dougherty et al., 2023). Secondly, our findings highlight the relevance of experiences over time, or learning effects, on memories of episodes. This is particularly important as this prompted us to take into account the interplay between learning (Xue et al., 2010) and sequence memories in future studies of learned order (Manns et al., 2007).

In the theoretical realm of temporal context, classical temporal context models (TCMs) have been influential in understanding episodic memory (for reviews, see Sederberg et al., 2008; Polyn et al., 2009); however, there have been suggestions that the TCMs have several notable oversimplifications. One of them concerns that the neural mechanism by which temporal context could be recovered has been unspecified in these models (Lohnas and Howard, 2024). The need for mechanistic accounts for retrieval of a temporal context has become increasingly relevant following discoveries of temporal context cells and their Laplace transform of time in the entorhinal cortex (Tsao et al., 2018; Bright et al., 2020) and the precuneus (Zuo et al., 2023), “time cells” in the hippocampus (Pastalkova et al., 2008; MacDonald et al., 2011), entorhinal cortex (Umbach et al., 2020), and cortical regions (Jin et al., 2009; Tiganj et al., 2017; Cruzado et al., 2020), as well as cells sensitive to both space and time (Kraus et al., 2013). In light of this body of evidence for the connection between temporal context representation and neurophysiology, our findings on the differential involvements of theta *P*_episode_ and gamma *P*_episode_ should help inform and augment the neurobiological account of temporal context retrieval for current TCMs.

Another important contribution is that we demonstrated an interplay between theta oscillations and learning for temporal order memory processes. During initial stages of learning, theta events are biased toward forward retrieval in the cued recall of forward/backward sequences. As learning occurs, the hippocampal theta episodes facilitate experience-dependent changes for directionality in retrieval. This evidence on how human hippocampal theta *P*_episode_ are implicated in learning suggests an important link between theta oscillations and how they incorporate learning and experiences toward modulating our episodic memory representation (Pacheco Estefan et al., 2021). As evidenced by earlier studies, the hippocampal system is critical to enable normal learning of temporal structure of events (Kwok et al., 2015), by bridging spatial information across delay (Naya and Suzuki, 2011; Sakon et al., 2014) and/or accumulating a history of spatial relationships (Forcelli et al., 2014; Rueckemann and Buffalo, 2017). To relate our findings with the broader animal literature, our results echo with models stipulating a common neural substrate underlying relational encoding encompassing both memory and navigation (Howard and Eichenbaum, 2015). The temporal order memory task here with two spatial directionality is a hybrid of both abstracted space and memory representation.

We performed a corresponding analysis using gamma oscillations and we found no such learning effects for gamma activity. There was a main effect of directionality in gamma *P*_episode_ rates, where forward trials had consistently higher *P*_episode_ across all stages. In free call paradigms, there is a higher probability of successively recalling items in a forward versus backward direction, where for a given recalled item, subsequently recalled items are more likely to have been encoded after (rather than before) the first-recalled item (Polyn and Kahana, 2008; Polyn et al., 2009). This forward asymmetry bias arises because a given item becomes part of the temporal context for succeeding items and serves as a memory cue. In our navigational case, a forward-cued condition, with it reinstating the sequence of items in an identical direction as encoding, would have reinstated more item representations within the sequence, thereby resulting in a strengthened temporal context upon subsequent memory test. Since gamma activity is involved in organizing and temporally segmenting the representations of different items (Pesaran et al., 2002; Howard et al., 2003), this might explain why gamma *P*_episode_ rates were consistently higher for forward than backward trials. The stability of gamma *P*_episode_ rates across all stages suggests that the gamma correlates were not affected by the theta-mediated “learning” we observed here and is consistent with the initial performance advantage before any theta-mediated “learning” occurred (Chan et al., 2009; Yang et al., 2013; Liu et al., 2014; Liu and Caplan, 2022). Moreover, by considering the dynamics between theta phase and gamma power, we found significant theta–gamma coupling during the memory task and that there is a numerically stronger coupling for the backward condition in the late stage. These theta–gamma cross-frequency coupling results imply a putative functional role of gamma in these memory processes.

Our findings on theta band activity carry conceptual relevance to the wider literature when considered under a recent framework on human action control (Beste et al., 2023). We have identified some conceptual relations between theta band activity in action control research and theta band activity during memory retrieval in hippocampal structures. According to the Binding and Retrieval in Action Control (BRAC) framework (Frings et al., 2020), action control is governed by two separate processes, namely, integration of event-files and retrieval of such event-files. This notion is applicable to learning and memory research too, where the past (event-file binding) can shape and modulate current behavior (via retrieval of event-files). For instance, in the current task the sequential structure of the item presentation is the event-file coding process, whereas the memory stage resembles the retrieval part of the event-file management dynamics. As stipulated by the BRAC model, the theta band activity and/or by proxy theta event frequency (theta *P*_episode_ rate) should support both binding and retrieval processes. In the memories of temporal order, both theta and gamma band activity are implicated (Roux and Uhlhaas, 2014). The synchronization of hippocampal theta and gamma band activity revealed by our phase–amplitude coupling analyses is linked to a theta–gamma mechanism in episodic memory (Lisman and Jensen, 2013; Hanslmayr et al., 2016) and more broadly event-file binding (Beste et al., 2023). The implication of such experiential/incidental learning should be pursued in a broader context covering other cognitive processes such as cognitive control (Takacs and Beste, 2023).

Sustained theta and gamma oscillations are respectively involved in the learning process for backward retrieval and forward asymmetry bias during temporal order memories. By highlighting that theta activity reflects the learning process during episodic experiences of order memory, this study helps elucidate the electrophysiological mechanisms underlying the interplay between learning and spatiotemporal sequence memories upon retrieval in the hippocampus.

## Data Availability

Raw electrophysiological data, analysis code, and processed data supporting the conclusion of this study are available here: https://drive.google.com/drive/folders/1HDtdrQd-F1vVVz0NdXaFZyYjkrne98Gn.
